# Detection of Depressive Symptoms in College Students Using Multimodal Passive Sensing Data and Light Gradient Boosting Machine: Longitudinal Pilot Study

**DOI:** 10.2196/67964

**Published:** 2025-06-03

**Authors:** Jessica L Borelli, Yuning Wang, Frances Haofei Li, Lyric N Russo, Marta Tironi, Ken Yamashita, Elayne Zhou, Jocelyn Lai, Brenda Nguyen, Iman Azimi, Christopher Marcotullio, Sina Labbaf, Salar Jafarlou, Nikil Dutt, Amir Rahmani

**Affiliations:** 1 Department of Psychological Science University of California, Irvine Irvine, CA United States; 2 Department of Computing University of Turku Turku Finland; 3 Department of Educational Sciences University of Genoa Genoa Italy; 4 Department of Psychology University of Southern California Los Angeles, CA United States

**Keywords:** depression, college students, emerging adulthood, machine learning, passive sensing

## Abstract

**Background:**

Depression is the top contributor to global disability. Early detection of depression and depressive symptoms enables timely intervention and reduces their physical and social consequences. Prevalence estimates of depression approach 30% among college students. Passive, device-based sensing further enables detection of depressive symptoms at a low burden to the individual.

**Objective:**

We leveraged an ensemble machine learning method (light gradient boosting machine) to detect depressive symptoms entirely through passive sensing.

**Methods:**

A diverse sample of undergraduate students (N=28; mean age 19.96, SD 1.23 y; 15/28, 54% women; 13/28, 46% Latine; 10/28, 36% Asian; 4/28, 14% non-Latine White; 11/28, 4% other) participated in an intensive longitudinal study. Participants wore 2 devices (an Oura ring for sleep and physiology data, and a Samsung smartwatch for physiology and movement data) and installed the AWARE software on their mobile devices, which collects passive sensing data such as screen time. Participants were derived from a randomized controlled trial of a positive psychology mobile health intervention. They completed a self-report measure of depressive symptoms administered weekly over a 19- to 22-week period.

**Results:**

The light gradient boosting machine model achieved an *F*_1_-score of 0.744 and a Cohen κ coefficient of 0.474, indicating moderate agreement between the predicted labels and the ground truth. The most predictive features of depressive symptoms were sleep quality and missed mobile interactions.

**Conclusions:**

Findings suggest that data collected from passive sensing devices may provide real-time, low-cost insight into the detection of depressive symptoms in college students and may present an opportunity for future prevention and perhaps intervention.

## Introduction

### Depression in College Students

Depression is the foremost contributor to global disability [1]. Longitudinal studies show that symptoms of depression typically begin in a person’s 20s to early 30s [2]. In recent years, college students’ mental health has worsened, with major depression rising disproportionately within this population [3-5]. College students assessed during the COVID-19 pandemic witnessed a 300% increase in the risk of developing depressive disorders as compared to the previous 8 years [6]. Given the cascading mental health and cognitive consequences of depressive symptoms [7,8], there is an urgent need for early detection of depression in college students to inform prevention and intervention efforts. The presence of depressive symptoms is a key indicator of risk for full-blown clinical depressive syndromes [9]. Consistent with a developmental psychopathology framework [10], this study examined the presence of depressive symptoms within a community sample of college students to develop and test an algorithm to predict depressive symptoms, and hence, risk for depressive disorders.

### The Biopsychosocial Model of Depression Risk

**Figure 1:**
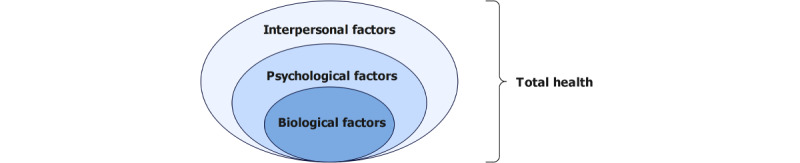
Conceptual model underpinning the use of multimodal real-world monitoring data to predict depressive symptoms.

The biopsychosocial model provides an integrative perspective that underscores the roles of different risk factors in predicting depression risk [11]. This model captures the complexity of individuals’ environments, acknowledging that everyone’s health is affected by a variety of biological, psychological, and social or interpersonal factors that coexist (Figure 1). Examining these factors in isolation provides an incomplete view of mental health. Thus, the central goal of this study was to use multiple indicators of functioning, including biological, psychological, and social factors, to predict depressive symptoms.

In support of the key tenets of this model, certain psychophysiological habits (eg, sleep and physical activity), along with specific behavioral factors associated with the lifestyle of emerging adults (eg, frequent use of electronic devices and mobile phones), substantially impact well-being both physically and mentally. These habits, particularly the use of electronic devices, are closely linked with depression and may act as predisposing factors [12], particularly among college students. In the sections that follow, we review 3 factors that have strong evidence for their association with depression within college students.

### Sleep and Depression Risk

Sleep quality has emerged as a reliable predictor of mental health among college students [13-15]. Individuals who fall asleep easily and maintain uninterrupted sleep exhibit lower levels of depression [16]. Conversely, poor sleep quality has emerged not only as a strong predictor of depression but also as a consequence of it [14,17], revealing a cyclic interplay between sleep patterns and mental health. Greater depressive symptoms in adolescence are associated with subjective and objective sleep difficulties in emerging adulthood; furthermore, these sleep difficulties are related to more depressive symptoms in emerging adulthood [18]. The importance of high-quality sleep for mental health was made particularly salient during the COVID-19 pandemic, when sleep disturbances increased among college students due to heightened stress, uncertainty, and disruptions to daily routines [12]. This reciprocal relationship highlights the importance of using objective measures to assess sleep quality and its effects on mental health, as these measures can provide valuable insights into sleep duration, efficiency, and fragmentation [19]. Considering these findings, investigating objective sleep quality as a predictor of depression is critical.

### Smartphone Use and Depression Risk

The vast majority (97%) of college students own a smartphone and use it daily [20]. Studies identify a bidirectional and reciprocal relationship between the quality and quantity of smartphone use in college students with mood [21,22]. Considering the function and motivation behind smartphone use can be useful in making sense of these connections [23]. Smartphones can be used in ways that promote mental health, but they can also be used in ways that are destructive or harmful. When smartphone use becomes intense, compulsive, or indiscriminate, such as when smartphones are used to disengage from or avoid aversive emotions [19,24], depressive symptoms may arise. Although research has predominantly focused on the tolls of smartphone use on health [21,22], the prevalence of smartphones and their integration into daily social life have also motivated research into the potential harms of disengagement from smartphones. For instance, Elhai et al [12] found that higher levels of depressive symptoms were associated with lower social smartphone use in adults, such as lower use of social media for fostering relationships or staying in contact with people. To the best of our knowledge, the links between socially disengaged smartphone use and depressive symptoms have not been explored in college students. Furthermore, we contend that social isolation may also manifest through other dimensions of smartphone use (eg, beyond social media use), such as not answering calls or texts, and that this behavior may be bidirectionally connected with depressive symptoms. The more depressed students feel, the less they use their phone, and the more socially disengaged they feel, the less they have the urge to use it to interpersonally interact. Moving forward, it is necessary to expand our knowledge regarding the frequency of social interactions, assessing the amount of engagement between college students and their smartphones in their daily life (ie, the rate of messages and calls) to better understand the connection with depressive symptoms. This study builds upon existing research by exploring these questions in college students and focusing on additional indicators of smartphone engagement or disengagement.

### Physical Activity and Depression Risk

Physical activity is a robust predictor of mental health and well-being in college students [25,26]. More physical activity in college students predicts lower anxiety, depression, and stress, as well as higher self-esteem, and overall improved mental health [24,27-31]. Physical activity decreases across the transition from adolescence to young adulthood, particularly for female individuals [27,32], which is concerning given its link with mental health. Importantly, physical activity is a modifiable behavior [33], making it an ideal target for intervention [24,34]. Particularly during the COVID-19 pandemic, physical inactivity emerged as a powerful predictor of depression, when options for remote learning reduced the need for movement and promoted a sedentary lifestyle [35,36]. Objective assessments of physical activity have the advantage of providing accurate documentation of behavior that may be difficult to remember or report. A recent meta-analysis found that objectively measured indices of physical activity are associated with depression risk [37]. Multiple indices of objectively-assessed physical activity, such as time spent being stationary [13,38], energy consumed [39], and number of steps taken [40], can be unobtrusively obtained from devices, providing a source of data that avoids some of the pitfalls of self-report measures of physical activity.

### Leveraging Machine Learning Methods

The use of machine learning (ML) methods with sleep, smartphone use, and physical activity data may offer unique advantages and insights in the detection of psychopathology. Among the array of ML approaches, classification tasks stand out for their capacity to map attributes (representing features of data instances) to a designated label, such as the presence of depression. Classification tasks remain the most used ML method in the diagnosis and detection of mental illnesses. A light gradient boosting machine (LightGBM) [41] is a form of supervised learning algorithm that offers state-of-the-art performance while remaining highly efficient. Built on a decision-tree architecture, LightGBM uniquely incorporates gradient-based information, allowing it to handle large-scale datasets while remaining model-efficient and using little memory.

While research applying and advancing ML methods to detect psychopathology is still in its early stages, there has been a surge of such research in recent years. Studies have applied various ML algorithms, encompassing different classes (ie, traditional ML and deep learning) and types (ie, supervised and unsupervised learning) of ML approaches. In addition, existing studies have also leveraged diverse types (ie, physiological, semantic, acoustic, or facial feature) of data [42-44]. These studies suggest some common features associated with depression, such as acoustic volumes (eg, lower pitch associated with more severe depression) [45], social media use (higher problematic social media use associated with more severe depression) [46], combined facial features (eg, the movement of pupil and mouth) [13]. Notably, 1 limitation of many existing studies is that data were collected from highly controlled laboratory settings and may not accurately reflect individuals’ day-to-day behaviors [47]. Combining LightGBM with wearable device data collection can offer precise and individualized detections of depression from data gathered in naturalistic settings. In addition, explainable artificial intelligence methods can be used to analyze the relative impact of each variable within the collected data on depression detections.

### This Study

Building upon previous work [48-51], this study uses digital detection processes as an indicator of depressive symptoms risk. Depressive symptoms can fluctuate over time, and accurately capturing depressive states may necessitate a fine-grained assessment methodology. We used 3 months of data from a randomized clinical trial conducted with a community sample of college students. We assessed depressive symptoms using a commonly used and reliable measure, the Patient Health Questionnaire-9 (PHQ-9) [52], to provide the most comprehensive assessment of depressive symptoms.

We pursued 3 aims. First, we sought to classify samples using a binary system, which we refer to as depressed or nondepressed (above or below PHQ-9 scores of 4, or mild depression) based on our data using ML techniques (aim 1). This aim involved examining the overall performance of the depression detection system. Meta-analyses on the efficacy of these models found that they predict the risk of depression based on both psychological and behavioral factors [40,53,54]. Using a confusion matrix to analyze the performance of the ML model, we hypothesized that the model would be able to differentiate between depressed and nondepressed participants. It is worth noting that although we use the terms *depressed* or *nondepressed* for ease of communication, it is important to recall that we examine these research questions in a community sample. Furthermore, we use a self-report questionnaire to assess depressive symptoms rather than clinician interviews of depressive disorders. Therefore, our use of these terms is shorthand, used for ease of communication, but should not be viewed as indicative of a clinical diagnosis.

Second, we examined depressive symptom detection across intervention versus control groups within our study (aim 2). The data used in this study were part of a randomized controlled trial examining a just-in-time mobile health intervention (ie, relational savoring) [55,56] designed to prevent loneliness (the study by Nguyen et al [unpublished data, 2024] provides an overview of the design of the intervention component of the study). Given that inclusion in the intervention condition could impact depressive symptoms as well as the relationship between contextual factors (ie, sleep, physiology, and behavior) with depressive symptoms, aim 2 involved examining depression detection separately across groups.

Third, we examined the features that contributed to the prediction of depressive symptoms (aim 3). We focused on the features that were important for the ML model to accurately identify indicators of depression. On the basis of previous literature, sleep quality is a variable that has repeatedly been found to be linked to depression [17,57,58]. From this, we developed our hypothesis that sleep quality measured through breathing patterns and movement would be the greatest indicator of depressive symptoms among physiological and behavioral measurements. Growing research has also identified earlier problematic mobile phone use as a predictor of depression later in development [59] and that call activity has been traced to indicate depression [60-62]. Therefore, we hypothesized that messaging and call frequency would be significant indicators among mobile activities when measuring depression. Finally, based on research linking physical activity to depression risk [37], we anticipated that low physical activity would be associated with depression risk. Exploratory analyses were conducted as a bottom-up approach where all potential passive sensing features were included in the same model to see which features emerged as most predictive.

The pursuit of these 3 aims combined to allow us to examine the utility of predicting depressive symptoms within a sample of college students using the LightGBM method, contributing to the knowledge base regarding ML and depression detection.

## Methods

### Participants

Undergraduate students between 18 and 22 years old were recruited through flyers and campus announcements at a large West Coast university in the United States. Participants met the eligibility criteria if they were fluent in English and used an Android smartphone with an operating system of 6.0 or higher (the study by Borelli [55] provides full details on participation eligibility). Exclusion criteria included anyone who is a parent, married, coming back to school after some time, aged >22 years, unable to speak or write English fluently, or currently meets criteria for depression. These exclusion criteria were included due to our desire to generalize our findings to the general population of college students. In total, 37 participants enrolled, with 10 withdrawing their participation over the course of the study (N=28; mean age 19.96, SD 1.23 years; 15/28, 54% women). Out of 28 participants, 13 (46%) identified as Latine, 10 (36%) as Asian, 3 (14%) as non-Latine White, and 1 (4%) participant as other. Participants’ demographic information is provided in Table 1.

**Table 1:** Participants’ demographic information (N=28).

Parameter			Participants
Age (y), mean (SD)			19.96 (1.23)
**Gender, n (%)**			
	Women	15 (54)	
	Men	13 (46)	
**Year level in college, n (%)**			
	First	4 (14)	
	Second	4 (14)	
	Third	12 (43)	
	Fourth	8 (29)	
**Race and ethnicity, n (%)**			
	Asian	10 (36)	
	Latine	13 (46)	
	White	4 (14)	
	Other	1 (4)	

### Ethical Considerations

The study was approved by the principal investigator’s institutional review board (HS 2019-5153) and informed consent was obtained from all participants before the inception of data collection. Depending on the completion of study components, participants were compensated between US $30 to US $660. We structured data handling into 3 phases: acquisition, transmission, and storage. Sensors were registered via media access control addresses, with secure and dynamic matching between users and devices. Access was restricted based on user level and duration, with University of California Irvine Office of Information Technology (OIT) conducting security assessments. For transmission, secure sockets layer, firewalls, and multifactor authentication secure communications were used, while OIT provided intrusion detection and monitoring. Local storage used encryption and key management, while cloud data were encrypted, with identifiers stored securely for report generation. OIT ensured secure transmission, backup, recovery, and disposal. We followed legal standards to protect personal data. The Anonymization and Reidentification Exchange Data Anonymization Tool removed identity traces, replacing them with local IDs. Sensitive details were abstracted, and personal data were locally processed, not stored in the cloud. Automated tools handle anonymized cloud data, with access limited to authorized Institutional Review Board–approved researchers.

### Procedures

All 28 participants began enrollment in the study between January and February of 2022 and participated for at least 4.5 months (19 weeks). After being deemed eligible, participants were scheduled for an in-person session where they were provided with wearable smart devices (Oura ring [Oura Health Ltd] and Samsung watch [Samsung Electronics Co, Ltd]) and directed to install study-related phone apps (AWARE) to record passive sensing data, as well as a different app to record daily and weekly surveys. After the research staff provided instructions on how to set up and use their devices, participants were informed on how to complete the questionnaires administered on a daily and weekly basis. Research assistants asked participants to wear or keep their devices with them as much as possible unless charging or engaging in any intense activity (eg, sports) that might damage the devices.

For subsequent procedures, we focus on those relevant to the study aims. Following the in-person laboratory session, participants completed a 6-week monitoring period, during which they wore the smart devices. On each Sunday during their participation in the study, participants completed weekly depression questionnaires. Passive sensing data (eg, geographic location, physiology, actigraphy, sleep, among others) were collected through the smart devices. After the 6-week monitoring period, participants were randomly assigned to 1 of 2 conditions—monitoring only, that is, the control condition in which participants continued as during the monitoring phase, and intervention condition, that is, the experimental condition in which participants received a mobile health intervention named relational savoring [53,54] delivered via their smartphone based on an algorithm. The central aim of the intervention was to examine whether delivery of this intervention reduced participant loneliness. Although the results of this intervention are outside the scope of this investigation, we refer interested readers to the reported findings present in the study by Borelli [55]. This randomized monitoring or intervention period phase lasted 4 weeks, followed by ≥9 weeks of monitoring only.

### Measures

#### Assessment of Depressive Symptoms

PHQ-9 [50] is a 9-item questionnaire and a standardized tool designed to screen, diagnose, monitor, and measure the severity of depressive symptoms, including mood, anhedonia, sleep, appetite, concentration, and suicidal ideation. The traditional questionnaire, which asks about depressive symptoms experienced over the past 2 weeks, was modified for use in this weekly context, for example, “over the last week, how often have you been bothered by any of the following problems: little interest or pleasure in doing things?” The PHQ-9 has robustly demonstrated validity and reliability across diverse populations, including US college students, which was the targeted population in this study [63]. While this measure has not been specifically validated with weekly administration, it has been widely used in published ecological momentary assessment–related studies for weekly administration, for example, in the study by Nickels et al [64] and with varying frequencies (eg, 3 times per day) [65], demonstrating appropriateness to be used in repeated measurements.

Items were rated on a 4-point Likert scale (from 0=not at all to 3=nearly every day) and summed, where higher scores indicated higher levels of depression. As expected for the current sample, depressive symptoms were relatively low across the study period (mean 4.90, SD 4.25). Thus, for this study, to address our aims of predicting depressive symptomatology and identifying the features most predictive of these symptoms, we created a dichotomous variable using a clinical cutoff: participants with scores >4 were identified as being in the “follow-up needed” group, whereas participants with scores of ≤4 were identified as being in the “none-minimal” group. Internal consistency in this sample was good (α=0.83). As described earlier, participants completed this measure weekly; participants were monitored throughout the assessment with supervision from the first author, who has clinical training and expertise. After 3 months of monitoring, there were a total of 355 valid PHQ-9 submissions and 4 missed submissions. The valid submissions were used as the depression labels in the subsequent analysis.

#### Passive Sensing of Health, Sleep, and Behavior

To capture an accurate depiction of participants’ daily physical habits, sleep, and health, we fitted participants with the Oura ring and Samsung Gear Sport smartwatch and downloaded the corresponding Oura and Samsung Android mobile apps. The Oura ring assesses sleep quality by measuring sleep duration, average heart rate (HR) during sleep, and HR variability (HRV) during sleep [66]. It also collects physical activity parameters, including energy consumption and moving steps. In addition to sleep and physical activity data, the Samsung Gear Sport smartwatch collected raw photoplethysmogram (PPG) signal using a green light LED sensor for 12 minutes every 2 hours, from which daily HR and daily HRV were extracted [67].

The watch feature engineering process involved extracting HR and HRV measures from PPG data. To extract the HR and HRV features, the PPG signals were divided into nonoverlapping 5-minute segments. Each segment was processed individually using the pipeline described in the Data Preprocessing section. The features were extracted from the detected systolic peaks. The full list of extracted watch features is included in Table S1 in Multimedia Appendix 1.

The data collected from the smartwatch were preprocessed with a developed PPG preprocessing pipeline [68]. The pipeline consisted of 3 stages: signal quality assessment (SQA), signal reconstruction, and PPG peak detection. The SQA stage involved the classification of PPG signals into 2 categories, namely “clean” and “noisy” [69]. After the SQA stage, short-term “noisy” segments, which lasted for less than 15 seconds, were reconstructed using a generative adversarial network model [70]. The generative adversarial network–based model consists of a generator and a discriminator. The generator learned the representative features of clean PPG signals, while the discriminator distinguished between generated and original signals. This adversarial process improved the model’s performance. The trained generator was used to reconstruct the distorted PPG signals. Subsequently, a trained dilated convolution neural network was used to detect systolic peaks [71] and interbeat intervals. Finally, HR and HRV-related features were extracted from the detected interbeat intervals information.

The Oura ring feature engineering process involved 2 categories: daily features and nondaily features (the full list is provided in Table S2 in Multimedia Appendix 1). The daily features were provided directly from the Oura ring with 1 value per day. The nondaily features collected 1 value every 5 minutes. The daily slope, intercept, SD, and mean from the 5-minute measures were extracted to obtain a daily representation for these nondaily features. The Oura ring’s PPG sensors captured the characterization of daily sleep and activity patterns.

#### Mobile Activity

The AWARE app passively collected biometric data and logged daily routines [72]. Collected data included movement (ie, steps and exercises), social relationship information (ie, amount of time spent with other people based on audio detection and proximity), daily rhythms (ie, routines such as going out vs staying in one’s home based on location), and phone interactions (ie, texting, calling, and app browsing). The connection between the AWARE app and research servers were encrypted to ensure the privacy of participants’ data. The AWARE feature engineering process involved the feature extraction from calls, messages, notifications, screen activities, and locations from the participants’ smartphones. The full list of AWARE-related features is demonstrated in Table S3 in Multimedia Appendix 1.

The phone call–related features in AWARE were obtained from 4 types of call events: incoming calls, outgoing calls, missed calls, and voicemail. Within a specified time window of 5 days, the total duration and counts of each call event type were calculated. For the message and notification features in AWARE, 2 types of event notifications (ie, received messages and sent messages) were calculated; each event type was summed during the 5-day time window.

To obtain the participants’ mobile phone use patterns, 4 types of screen use events were analyzed: screen on, screen off, screen lock, and screen unlock. From these features, screen activity use was calculated through the sum of these events over the preceding 5 days.

For location data, the latitude and longitude data of meaningful addresses from the Google Map application programming interface [73] were extracted in terms of variance of latitude, variance of speed, mean speed, number of places visited, home-stay duration, outdoor-stay duration, mean outdoor-stay duration, SD of outdoor-stay duration, the type of place with the longest duration (excluding home), and total travel distance over a 5-day window.

### Data Analytic Plan

**Figure 2:**
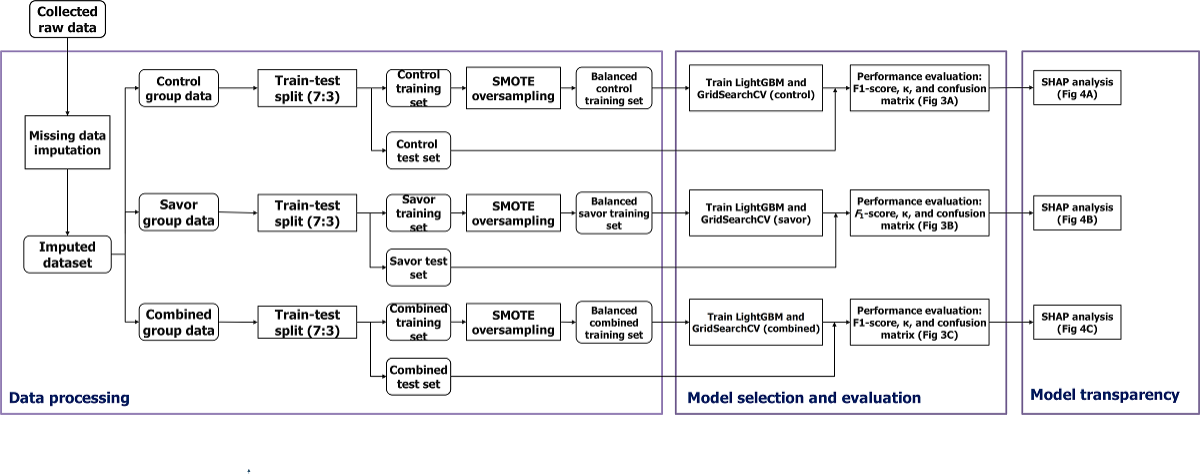
Data analytic flowchart. LightGBM: light gradient boosting machine; SHAP: Shapley Additive Explanations; SMOTE: synthetic minority oversampling technique.

The overall data analytic process is illustrated in Figure 2. Given the study design, the data analysis was conducted separately for the control group, the savor group, and the combined dataset (control+savor). The data analysis process includes data processing, model selection, evaluation, and model transparency.

### Data Preprocessing

Before building the ML model, several data preprocessing steps were applied to ensure data quality and suitability for model training. The aforementioned PPG preprocessing pipeline [64] was applied to reconstruct the poor-quality PPG signals with short duration and remove the unreliable PPG signals with long duration. The missing values of the dataset were then imputed with the nearest previous value of the time series. The synthetic minority oversampling technique (SMOTE) was used to preprocess the resulting imbalanced training dataset and will be described subsequently.

### Missing Value Imputation

In the longitudinal monitoring study, multiple factors resulted in missing values, including human-related issues (ie, forgetting to charge the devices or removal of the wearables due to daily activities) and technical issues (ie, interruption of data collection due to server congestion and permission allowance on the phone). Such missing data were classified as missing completely at random, as its occurrence was independent of the depression scale [74].

To quantify the extent of missing data, we computed the missing data percentages across participants. The overall missing rate varied across data sources, with smartphone-based passive sensing data showing an average missing rate of 16% and wearable-derived physiological data missing 11% of values.

Missing values were handled using the nearest-previous imputation method. This technique replaces the missing values with the nearest previous nonmissing value in the same feature column. We selected this method due to its ability to preserve temporal continuity in time series data, which is crucial for longitudinal analysis. Alternative imputation methods such as mean or median imputation were considered but deemed unsuitable as they might distort temporal structure of the data. Advanced ML-based imputations (eg, k-nearest neighbors or multiple imputation) were not applied to avoid introducing artificial patterns or biases in the data [75]. This method was applied to continuous physiological and smartphone use data, as these signals exhibit temporal dependencies where the previous observation is often the best estimate in short-term missing data scenarios. For ecological momentary assessment–related responses, missing values were not imputed to maintain the integrity of self-reported measures.

### SMOTE Assessment

The dataset used for depression detection was imbalanced, with a larger proportion of nondepressed participants compared to depressed cases. To address this issue, the SMOTE was used to generate synthetic training samples for the minority class. The SMOTE technique created new synthetic samples by oversampling a random sample in the minority class and its k-nearest neighbors in the feature space [76]. In the original training set, there were 126 and 109 instances in “nonminimal” class and “follow-up needed” class, respectively. After performing the SMOTE technique, there were 126 samples in both the “nonminimal” class and the “follow-up needed” class.

### Model

LightGBM was chosen as the ML algorithm to detect the individuals as depressed or nondepressed, considering the high dimensionality of the dataset comprising 104 features. LightGBM is a gradient boosting framework that uses tree-based learning algorithms to build predictive models. By using Exclusive Feature Bundling technique, LightGBM is best suited to effectively handle large numbers of features [39].

### Performance Evaluation

The *F*_1_-score is the harmonic mean of precision and recall, providing a balanced measure of a model’s performance [77]. The *F*_1_-score is calculated where the precision is the proportion of true positives among the sum of true positives and false positives. The recall is the ratio of true positives to the sum of true positives and false negatives as defined in equation 1.

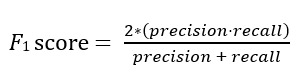



Cohen κ measures the agreement between the model’s predictions and the ground truth [78]. The variable *p*_0_ is the observed agreement between the model’s predictions and the ground truth, while the variable *p*_e_ is the expected agreement by chance, which is the marginal probabilities of agreement for each class. This relationship is calculated through equation 2.

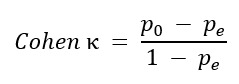



## Results

### Descriptive Statistics and Correlations

We conducted separate analyses for the control group, savor group, and the combined dataset (control+savor). For each group, the dataset was split into 70% training and 30% testing, with 3-fold cross-validation applied during training for hyperparameter tuning. The final test dataset contained 27 depressed and 23 nondepressed instances for the control group; 22 depressed and 30 nondepressed instances for the savor group, and 41 depressed and 60 nondepressed instances for the combined dataset. These instances were derived from 28 unique participants.

### Aim 1: Overall Performance of Depressive Symptom Detection

The overall performance of the depression detection model with the observations from 28 participants (ie, combined group) is shown in Table 2. Given that this study was conducted within the context of an intervention study, we present our findings separately by group (control and intervention) as well as combined across groups. To evaluate model fit, we compared the detected labels (ie, the label predicted by the detection model) and the ground truth (ie, the depression severity reported on weekly PHQ-9 questionnaires). Our model achieved an *F*_1_-score of 0.744 and a Cohen κ coefficient of 0.474. A higher *F*_1_-score generally represents better detection capability, and our model achieved a moderate score, and the Cohen κ coefficient [79] indicated a moderate agreement between the predicted labels and the ground truth.

**Table 2:** Overall performance of the depressive symptom detection for control, intervention, and combined models.

Group	True positive	True negative	False positive	False negative	Accuracy	Precision	Recall	*F*_1_-score	Cohen κ
Control	18	14	9	9	0.64	0.667	0.667	0.667	0.275
Intervention	14	23	7	8	0.712	0.667	0.636	0.651	0.405
Combined	30	45	15	11	0.743	0.667	0.732	0.698	0.474

**Figure 3:**
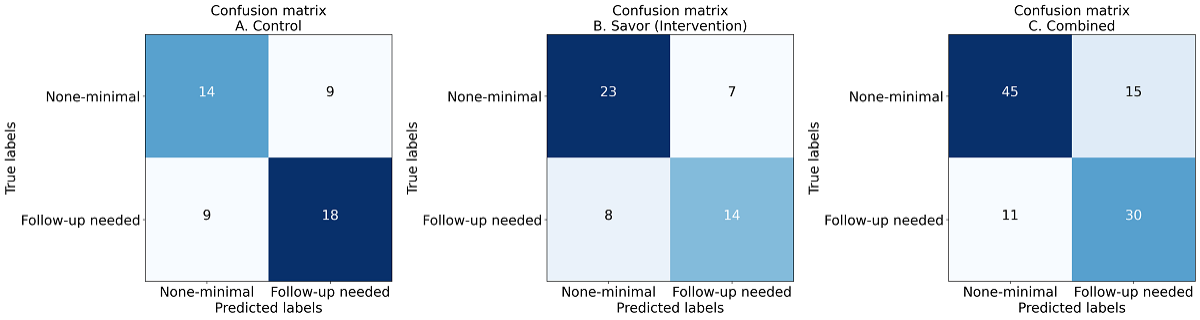
Confusion matrix of depressive symptom detection model performance for (A) control, (B) savor (intervention), and (C) combined (ie, control and intervention) groups.

The confusion matrix of the detection results is shown in Figure 3. Our model successfully detected the 45 out of 60 “none-minimal” participants (ie, those who scored between 0-4 on the PHQ-9) and 30 out of 41 “follow-up needed” participants (ie, those who scored between 5 and 9 on the PHQ-9). These results indicate that our model was able to accurately assess the depression level of the individuals based on their physiological and behavioral data, achieving an *F*_1_-score of 0.70, a sensitivity of 0.73, a precision of 0.67, and an accuracy of 0.74.

The full confusion matrices for the combined, control, and intervention groups are summarized in Table 2, and the detailed confusion matrices are provided in Figure S1 in Multimedia Appendix 2.

### Aim 2: Detect Depressive Symptoms Across Control, Intervention, and Combined Participant Groups

In this study, we evaluated the efficacy of depression detection models across different participant groups, specifically the control group, the intervention group, and the combined dataset encompassing both groups. In total, 3 LightGBM models were trained separately for the control group, the intervention group, and the combined group. The performance metrics for these models were assessed using the *F*_1_-score and Cohen κ. The control group model yielded an *F*_1_-score of 0.640 and a Cohen κ of 0.275, indicating moderate detection performance and agreement. In contrast, the intervention group, which received targeted just-in-time interventions during the second month, demonstrated improved detection with an *F*_1_-score of 0.711 and a Cohen κ of 0.405, reflecting better model accuracy and substantial agreement. The combined model, integrating both control and intervention group data, achieved the highest performance, with an *F*_1_-score of 0.744 and a Cohen κ of 0.474. The most plausible explanation for these differential findings is that the combined model provided more sample size, enabling greater predictive capacity. Differences between the relational group and the control group may be explained by the relational intervention reducing variability among participants—in other words, all participants received an intervention, enabling greater predictability of symptoms.

### Aim 3: Explainability and Feature Importance Analysis

**Figure 4:**
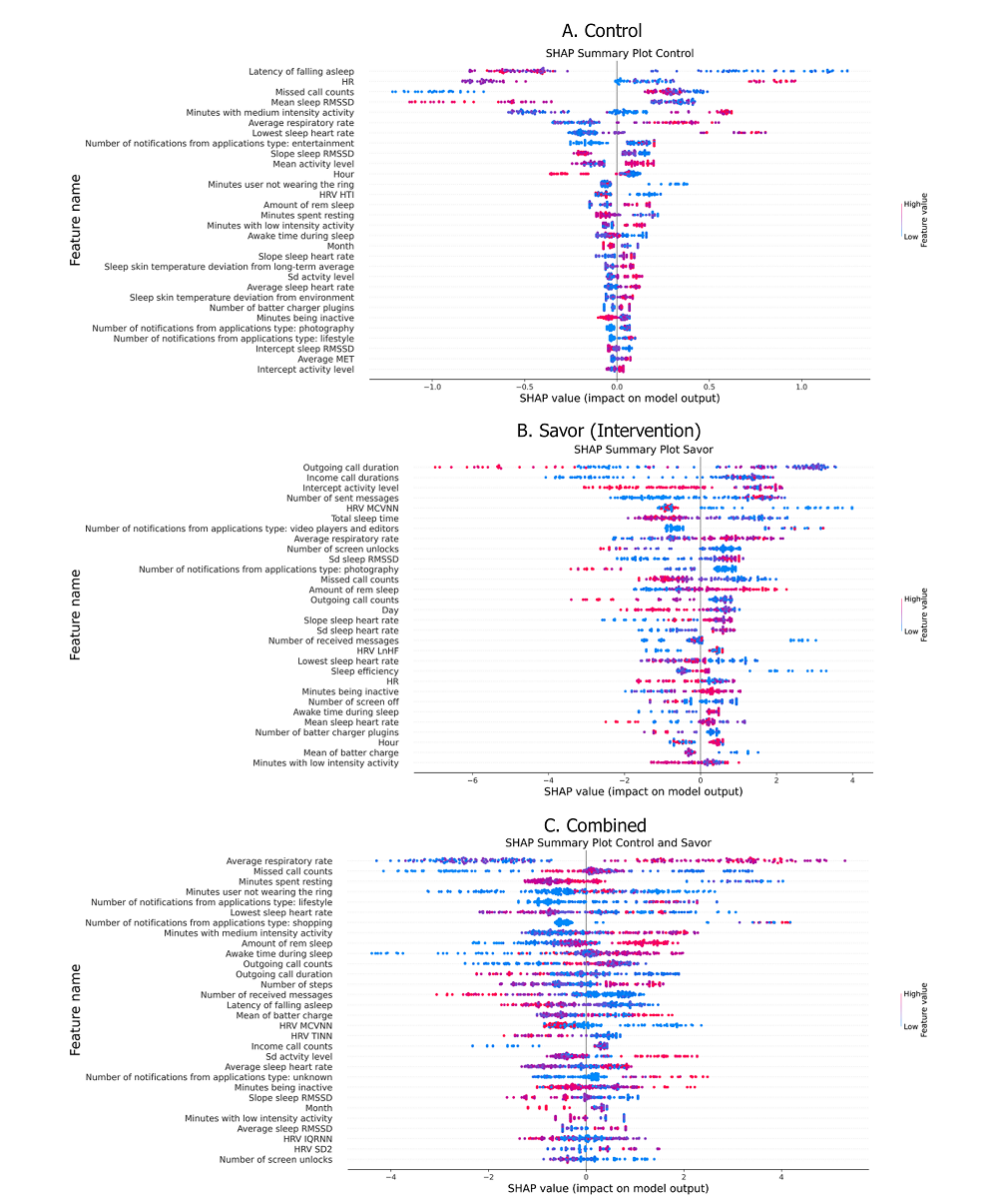
Shapley Additive Explanations (SHAP)–based explainers of top 30 features for (A) control, (B) intervention, and (C) combined (ie, control and intervention) groups’ depressive symptom detection. HR: heart rate; HRV: heart rate variability; HTI: heart rate triangular index; IQRNN: IQR of normal-to-normal intervals; LnHF: ratio of low frequency to high frequency; MCVNN: mean consecutive variation of normal-to-normal intervals; RMSSD: root mean square of successive differences; TINN: triangular interpolation of normal-to-normal histogram.

To enhance the interpretability of our depression detection model, we used Shapley Additive Explanations (SHAP) to analyze feature importance and their effects on the model’s outputs [80]. SHAP values provide a unified measure of feature contribution by calculating the change in the model’s prediction when a feature is added or removed. This approach ensures a consistent and reliable determination of each feature’s impact. By calculating SHAP values, we can ascertain how each feature influences the prediction. The x-axis in our SHAP summary plots (Figure 4) represents the SHAP value, indicating the impact of each feature on the model’s output: positive values suggest a feature pushes the prediction toward depression, while negative values push it away. Meanwhile, the y-axis lists the feature values, with each point color-coded to show the actual feature value (ie, low to high).

Figure 4 presents the top-30 features obtained by SHAP analysis for the control group, intervention group, and the combined dataset. For the control group, the 5 most important features for the detection were sleep latency, average HR, missed call counts, average sleep root mean square of successive differences, and minutes with medium activity intensity. Specifically, for the most predictive feature, sleep latency, lower values were associated with a higher likelihood of depression detection.

In the intervention group, the 5 key features driving the model’s predictions were outgoing call duration, incoming call duration, the intercept of daily activity level regression (indicating an individual’s initial activity level upon waking), number of sent messages, and mean consecutive variation of normal-to-normal intervals. For the most predictive feature, outgoing call duration, both extreme high and low values predicted a lower likelihood of depression detection.

For the combined group, integrating data from both control and intervention participants, the most influential features were average sleep breathing rate, missed call counts, duration of resting minutes throughout the day, nonwear time of wearable devices, and the number of notifications from applications in the “lifestyle” category [81] (eg, Samsung Wallet/Pay and SmartThings). In this group, the most predictive feature, average sleep respiratory rate, demonstrated that higher values were associated with a greater likelihood of depressed detection.

## Discussion

### Main Findings

Our goal was to build upon previous work and investigate the utility of an ML approach for integrating mobile sensing data (ie, sleep, physiology, physical activity, and smartphone data) in the prediction of depressive symptoms as measured by weekly PHQ-9 questionnaires within a nonclinical sample of college students during the COVID-19 pandemic. Furthermore, we were interested in isolating the features that were most highly predictive of depressive symptoms to identify potential targets of prevention and intervention efforts. Our findings revealed that the ML model (LightGBM) was successful in differentiating participants in a low depressed and high depressed group, as indicated by 2 labels (“none-minimal” and “follow-up needed”), with a satisfactory *F*_1_-score. Furthermore, certain features were most strongly predictive of membership in the high depressed group, such as the average sleep breathing rate. We discuss these findings subsequently.

First, the data derived from the passive sensing devices and LightGBM yielded sufficient information to predict participants’ depression scores, measured weekly, with sufficient accuracy. The model in our study had an *F*_1_ statistic of 0.74, and a Cohen κ of 0.474, which indicates moderate agreement between the prediction and actual observations adjusted for random chance [73]. In interpreting these findings, it is important to note that we predicted group membership using a dichotomous grouping (“none-minimal” and “follow-up needed”). Group membership was based on a clinical cutoff that is meaningful [82,83]; people with scores >4 (minimal depression or greater) are more likely to experience consistent symptoms of depression, influenced by both length of symptoms as well as severity [84]. We created these groupings to increase the predictive power and clinical significance of our findings in the context of a small sample study. However, as we dichotomized the variable, we invited the trade-off that the ML findings do not reflect the various degrees of depressive symptoms.

Second, our study revealed that features differed in the degree to which they predicted depressive symptoms. We analyzed the features separately within the control and the intervention groups. Within the control (monitoring only) group, the strongest predictors of depression risk were sleep latency, average HR, missed call counts, average sleep root mean square of successive differences, and minutes with medium activity intensity. Within the intervention (relational savoring) group, the stronger predictors were outgoing call duration, incoming call duration, the interception of daily activity level regression, number of sent messages and HRV mean consecutive variation of NN intervals. When the groups were combined, the strongest predictors were average sleep breathing rate, number of missed calls, duration of resting minutes throughout the day, nonwear time of wearable devices, and number of notifications from apps in the “lifestyle” category (eg, Samsung Wallet or Pay). Notably, of the 5 most highly predictive features, 2 pertained to sleep (ie, average sleep breathing rate and total amount of wake time recorded during the sleep period), 2 pertained to smartphone use (ie, on-wear time of wearable devices and number of missed calls), and 1 pertained to physical activity (ie, duration of resting minutes). In other words, all 3 areas of inquiry were represented in the top predictors. In general, they were associated with depression in theoretically predictable directions—falling asleep quickly, breathing faster during sleep, and making many and very few calls were associated with a greater likelihood of depression. The findings are consistent with previous studies showing that objective measures of sleep [16], smartphone use and misuse [85,86], and physical activity [24] are all associated with depressive symptoms in college students. However, our study extends the existing literature by using wearable devices in everyday settings under free-living conditions. Most studies have relied on controlled laboratory environments, which may not capture the nuanced, real-world interactions of physiological and behavioral factors. Moreover, the long-term and fine-grained quantitative measurements obtained from wearable and mobile devices allow for a responsive monitoring of depression symptoms. By revealing the contributing factors related to depression for both the control group and the savor group, our study highlights the differential impacts of these variables depending on the intervention status.

Our findings suggest that it may not be possible to use a single source of information, such as a smartphone or wearable device, to predict depressive symptoms on college campuses. This underscores the need for personalized assessment of mental health, making the argument that many different indicators are important in predicting mental health outcomes [87-92]. Although this is exciting from a research perspective, it reduces the clinical utility of this work at the current time. A top predictor of depressive symptoms in the combined group, as well as in each subgroup, was derived from the Oura ring, which may suggest that of the data analyzed in this study, these may be the most useful or important in predicting depressive states in this population.

### Strengths and Limitations

The study is characterized by several strengths, including the use of a longitudinal design, a racially and ethnically diverse sample, and the incorporation of repeated depressive symptoms assessments. The inclusion of multiple passive sensing measures in the study is an additional strength that increases the generalizability of the findings and moves beyond previous studies on this topic. The fine-grained approach to understanding people’s experiences and behaviors helps to address the heterogeneity of symptomatology and depression to allow for better assessment of depressive symptoms. Furthermore, the study used a nonnested cross-validation procedure, which enhances the robustness and generalizability of the findings by making efficient use of the available data and reducing overfitting. Finally, participants in this study had high rates of adherence to study protocols—although some participants dropped out at the outset of the study, participants who continued with the study provided consistent data streams, increasing confidence in the conclusions we could draw from the study.

There are several limitations worth considering as well. This study was conducted with a community sample of college students rather than a clinical sample. Our goal was preventive in nature—to be able to detect depression risk before full-blown depression has manifested. On the basis of the assumption that subclinical levels of depressive symptoms confer risk for full-blown clinical depressive syndromes, we use depressive symptoms as a proxy here for risk for future depression. We urge readers to keep this in mind when interpreting these findings. To test whether the findings of this algorithm have predictive validity for the prediction of clinical levels of depression, a follow-up study would need to be conducted in which the study uses a clinical sample of participants who met criteria for major depressive disorder. The study would also benefit from the inclusion of diagnostic interviews at baseline and follow-up to assess for the presence of depressive disorders (rather than depressive symptoms).

Furthermore, the sample size was small, which considerably limits the generalizability of the findings. This study was conducted using a pilot study grant; given the technology, equipment, and staffing involved in this study, more funding would be required to support a study with a larger sample. The findings of the study indicate the importance of a personalized approach to assessment, which argues against reducing the number of data points, at least initially. However, it may be possible to increase the sample and reduce the cost by minimizing the measurement window. The creation of data repositories across research teams could facilitate larger-scale investigations with fewer resources.

An additional limitation involves our use of college students as participants. College students are an important population to target due to their high risk for mental health issues such as depression. However, their familiarity and comfort with technology and mobile devices distinguish them from other groups [93]. This restricts the generalizability of the findings. To have a better sense of the feasibility of this study’s design, it would be important to repeat this study among young adults who are not in college, as well as with older adult samples (eg, middle-aged and older adults) less familiar with technology.

Furthermore, despite being longitudinal, the study design is correlational, limiting our ability to make causal inferences. Our study used features collected 5 days before the PHQ-9 assessment, supporting the notion that passive sensing features indicate subsequent depression. However, it is possible that depressive symptoms in the previous week may influence the passive sensing indicators observed in the current week; therefore, confounding the direction of this relationship. As a result, the key indicators identified in this study cannot necessarily be considered predictors of depression. While separating the cause and effect in this relationship is extremely difficult, given the reciprocal and mutually reinforcing links between these indicators and depressive symptoms, identifying predictors of depressive symptoms is essential to pinpoint targets for prevention efforts. Furthermore, the current model did not consider interrelations between different contributing factors, for instance, sleep may influence exercise and exercise may influence sleep, both of which can in turn influence depression. In other words, our model assumed that each of these factors has independent rather than intersecting influences on depression, when in reality the interrelation between these factors may be much more complex and reciprocal. Furthermore, while SHAP-based explanations enhanced our understanding of the model’s internal decision process, these should be interpreted with caution. SHAP values reflect the contribution of each feature to the prediction within the model, and do not imply a causal relationship with depression symptoms. The SHAP framework is model-dependent and can be influenced by feature correlations, sampling variability, and model complexity. Future studies using larger and more diverse samples, along with causal inference techniques, are needed to validate the observed patterns. In this study, we use SHAP to enhance transparency and to generate hypotheses, rather than to make strong causal claims. In addition, analyses did not control for covariates that could be relevant to the questions of interest, such as BMI, socioeconomic status (which could be related to the type of mobile device used, namely Android versus Apple), travel (sleep routine), or psychological comorbidities, such as anxiety, which is highly related to depression in college students [94].

Given the difficulty in assessing depressed mood states, as well as the degree to which college students increasingly engage in life in an internet-based environment [95,96], the ability to predict mental health from passive sensing devices is of great utility. This investigation builds upon other studies that have adopted a similar approach of using mobile devices to collect real-world data in the service of predicting important outcomes [55,80,97], such as mental health. Although the current protocol as it was conducted does not have a high potential for widespread accessibility in that it involved multiple devices, multiple staff monitoring data collection, a multi-person computer science team to analyze the data, and a licensed psychologist to assess and provide mental health resources when participants reported elevated mental health symptoms, the ultimate vision for this work involves increasing access to care. Over the long term, we envision a scenario in which remote monitoring could reduce the need for mental health interventions or facilitate timely interventions by a mental health professional, thereby reducing costs and improving quality of care [98].

### Conclusions

This study offered an exciting glimpse into the potential of ML for predicting depressive symptoms among college students. Limitations notwithstanding, our study suggested the promise of multimodal passive sensing and ML to identify and respond to episodes of depressed mood. With the high global disease burden of depression and the widespread adoption of mobile and wearable devices, we hope this work can serve as a foundation for future studies that refine, streamline, and personalize our sensing and prediction paradigm, and that explore the utility of similar approaches with regard to other disorders in addition to depression. Ultimately, we hope this work culminates in the development of a monitoring system that can be implemented at scale. Such a system would offer an efficient, data-driven solution to mental health care and could improve depressive outcomes by helping connect individuals in need of mental health resources in a timely fashion, empowering them to proactively respond to challenges and maintain well-being.

## Appendix

Multimedia Appendix 1 Full list of features.

Multimedia Appendix 2 Confusion matrix for model performance.

## Abbreviations

HR: heart rate
HRV: heart rate variability
LightGBM: light gradient boosting machine
ML: machine learning
OIT: Office of Information Technology
PHQ-9: Patient Health Questionnaire-9
PPG: photoplethysmogram
SHAP: Shapley Additive Explanations
SMOTE: synthetic minority oversampling technique
SQA: signal quality assessment
