# Supplementary material for: Detection of Depressive Symptoms in College Students Using Multimodal Passive Sensing Data and Light Gradient Boosting Machine: Longitudinal Pilot Study
Source: JMIR Form Res. 2025 Jun 3;9:e67964. doi: 10.2196/67964 (PMC12174877; doi:10.2196/67964)
Supplement: Multimedia Appendix 2 [file formative_v9i1e67964_app2.docx]

Figure S1. Confusion Matrix of Depressive Symptom Detection Model Performance for (a) Control, (b) Savor (Intervention), and (c) Combined (i.e., Control and Intervention) Groups.

**
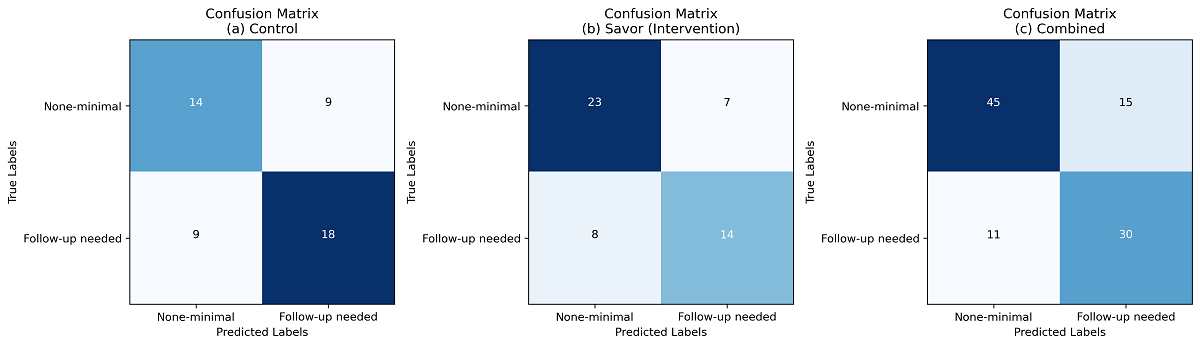
**
